# Neutrophil extracellular traps in atherosclerosis: current evidence and therapeutic potential of plant-derived metabolites

**DOI:** 10.3389/fphar.2026.1830396

**Published:** 2026-05-19

**Authors:** Jixin Li, Fengzhao Liu, Ruiling Zhou, Guanqi Fan, Shengnan Shi, Peili Wang

**Affiliations:** Chinese Academy of Traditional Chinese Medicine, Xiyuan Hospital, Beijing, China

**Keywords:** atherosclerosis, immunity, inflammation, neutrophil extracellular traps, neutrophils, plant-derived metabolites

## Abstract

Atherosclerosis is a chronic inflammatory vascular disease in which dyslipidemia, endothelial dysfunction, and maladaptive innate immunity jointly drive plaque initiation, progression, and rupture. Among the innate immune mechanisms involved, neutrophil extracellular traps (NETs) have emerged as important amplifiers of endothelial injury, macrophage activation, necrotic core expansion, and immunothrombosis. These findings suggest that restoration of NET homeostasis may help attenuate atherosclerotic progression. Accordingly, plant-derived metabolites have attracted increasing attention because some of them reduce NET-associated readouts in cellular and preclinical models. However, the current evidence base remains uneven. For many polyphenols and flavonoids, the reported anti-NET activity is derived mainly from simplified *in vitro* systems and may be confounded by pan-assay interference compounds (PAINS)-like assay interference. Therefore, statistically significant reductions in extracellular DNA, reactive oxygen species (ROS), or myeloperoxidase (MPO)-related signals should not be overinterpreted as evidence of selective NET inhibition or clinically relevant efficacy in humans. At present, most plant-derived metabolites should be regarded as hypothesis-generating candidates rather than validated therapeutics. This review summarizes the contribution of NETs to atherosclerosis, critically appraises the pharmacological and translational strength of the available literature on these metabolites, and outlines future directions based on orthogonal NET assays, disease-relevant models, pharmacokinetic grounding, and biomarker-guided clinical studies.

## 1 Introduction

Atherosclerosis is a systemic vascular disease characterized by plaque accumulation within the arterial intima. Multiple risk factors, including hypertension, diabetes, and smoking, contribute to its development. At advanced stages, the disease may lead to plaque rupture and subsequent adverse cardiovascular events (Zhu, 2026). Sterile inflammation is a key initiating event in atherosclerosis (Libby and Soehnlein, 2025; Mensah et al., 2025). Oxidized low-density lipoprotein (oxLDL) and cholesterol activate endothelial cells (Jiang et al., 2025; Lu-Chen et al., 2025). This activation promotes the recruitment of circulating monocytes into the subendothelial space, where they differentiate into macrophages and then into foam cells. As a result, lipid core formation and plaque growth accelerate, which are hallmark pathological features of atherosclerosis (Wang et al., 2025; Goncalves et al., 2026).

The chronic sterile inflammatory response in atherosclerosis is mediated by multiple immune cell types, including macrophages, T cells, dendritic cells, and neutrophils. These cells contribute to plaque progression and shape the local inflammatory microenvironment (Song et al., 2022). In addition to monocyte-derived macrophages, which have long been regarded as central to disease pathogenesis, neutrophils, the most abundant leukocytes in the circulation, are now recognized as important contributors to atherosclerosis risk (Mensah et al., 2025). In response to specific inflammatory and danger signals, neutrophils release neutrophil extracellular traps (NETs). NETs are composed of a DNA scaffold, histones, and granule proteins, and function as an innate immune defense mechanism that traps pathogens (Gao et al., 2025). Recent studies have shown that unstable plaques exhibit increased expression of myeloid differentiation protein-1 (MD-1), which may activate Toll-like receptor signaling and promote NET formation (Cao et al., 2024). NETs, in turn, increase the expression of endothelial adhesion molecules such as intercellular adhesion molecule-1 (ICAM-1) and vascular cell adhesion molecule-1 (VCAM-1). NETs also promote intraplaque neovascularization, thereby further compromising carotid plaque stability. In parallel, emerging evidence suggests that some plant-derived metabolites may modulate these processes and may therefore exert protective effects against atherosclerosis.

Accordingly, this review summarizes recent advances in understanding the role of NETs in atherosclerosis and discusses plant-derived metabolites that have been reported to regulate NET formation and attenuate atherosclerotic progression. This review aims to provide a reference for the prevention and treatment of atherosclerosis.

## 2 Data collection methods

We performed a comprehensive literature search in Web of Science, PubMed, EMBASE, and MEDLINE. The search strategy used both individual and combined keywords, including “neutrophil,” “neutrophil extracellular traps,” “plant-derived metabolites,” “traditional Chinese medicine,” “phytochemicals,” “atherosclerosis,” “thrombosis,” and “plaque.” We included relevant studies published up to 31 January 2026. The inclusion criteria were as follows: (1) original experimental studies, including *in vivo*, *in vitro*, *ex vivo*, or other preclinical studies, that investigated the effects of plant-derived metabolites, isolated components of traditional Chinese medicine, or specific phytochemicals on NET formation or NET-related pathways with plausible relevance to atherosclerosis; (2) studies that examined NET formation, release, degradation, or related signaling pathways and assessed their effects on endothelial inflammation and barrier dysfunction, lipid accumulation and plaque formation, plaque rupture, or thrombosis; and (3) peer-reviewed journal articles published in Chinese or English, as well as theses with full-text availability. The exclusion criteria were as follows: (1) studies involving crude extracts, compound preparations, or mixtures with undefined compositions; (2) studies that did not address NET-related mechanisms or showed limited relevance to the pathological processes of atherosclerosis; (3) review articles, meta-analyses, conference abstracts, commentaries, or studies without accessible full texts; and (4) studies with major methodological limitations, such as a lack of appropriate controls or poor reproducibility. During the critical appraisal, we specifically evaluated assay specificity, orthogonal validation of NET readouts, disease relevance of the experimental models, realism of the tested concentrations or doses, adequacy of controls, and translational distance from the model system to human disease. Based on this strategy and these criteria, we included 13 eligible articles, which yielded 14 metabolite-model study entries, in the critical synthesis.

## 3 Mechanisms of NET formation

NETs were first described by Brinkmann and colleagues as fibrillar network structures released by neutrophils into the extracellular space (Brinkmann et al., 2004). The process of NET formation is commonly termed NETosis (Liang et al., 2024; Kashif et al., 2025). Based on current evidence, NET formation can be broadly classified into three modes: suicidal NET formation, vital NET formation, and mitochondrial DNA (mtDNA)-dependent NET formation.

Suicidal NET formation is the most extensively studied mode and is generally considered a nicotinamide adenine dinucleotide phosphate oxidase (NOX)-dependent process. This process usually takes 2–4 h (Yipp and Kubes, 2013). Stimuli such as phorbol 12-myristate 13-acetate (PMA), cholesterol crystals, monosodium urate crystals, immune complexes, and microbial components can trigger this process (Jorch and Kubes, 2017). After receptors such as G protein-coupled receptors, Fcγ receptors, and complement receptors recognize these stimuli, intracellular Ca^2+^ levels increase (Huang et al., 2022). This increase activates protein kinase C (PKC) signaling (Hakkim et al., 2011), promotes NOX complex assembly, and enhances reactive oxygen species (ROS) generation (de Bont et al., 2018; Pieterse et al., 2018). Reactive oxygen species then facilitate the translocation of myeloperoxidase (MPO) and neutrophil elastase (NE) from cytoplasmic granules to the nucleus. Neutrophil elastase cleaves histones and promotes chromatin decondensation, whereas MPO further enhances chromatin relaxation. Ultimately, the nuclear membrane and plasma membrane rupture, which leads to NET release (Papayannopoulos et al., 2010).

In contrast, vital NET formation usually does not require neutrophil death and is often described as relatively NOX-independent (Yipp et al., 2012). In this process, neutrophils remain viable after NET release and retain certain immune functions (Zeng et al., 2025). Calcium ionophores, activated platelets, complement proteins, and microorganisms can induce vital NET formation (Papayannopoulos et al., 2010; Kenny et al., 2017). A common feature of this pathway is rapid Ca^2+^ influx, which activates peptidyl arginine deiminase 4 (PAD4) independently of NOX. Small-conductance calcium-activated potassium channel 3 (SK3) may also contribute to the regulation of Ca^2+^ influx in this context (Douda et al., 2015). After chromatin decondensation, nuclear material associates with histones and granule proteins and is packaged into vesicles derived from the nuclear envelope. These vesicles then move through the cytoplasm and fuse with the plasma membrane, thereby releasing chromatin-protein complexes into the extracellular space and forming web-like structures (Zeng et al., 2025; Stoiber et al., 2015). Mitochondrial DNA-dependent NET formation shares some features with vital NET formation. In many models, this process does not cause overt nuclear disruption, and neutrophils may retain partial functions such as chemotaxis and phagocytosis (Yousefi et al., 2009). This process appears to depend largely on mitochondrial reactive oxygen species (mitoROS) signaling (Reithofer et al., 2020). Mitochondrial reactive oxygen species may also influence Ca^2+^ influx, although the causal relationship remains incompletely defined and may vary across experimental models (Chen et al., 2021). At the disease level, mtDNA-containing NETs have been implicated in the progression of disorders such as systemic lupus erythematosus and trauma-related inflammation (Yousefi et al., 2009; Wang et al., 2015).

## 4 NETs promote the onset and progression of atherosclerosis

**FIGURE 1:**
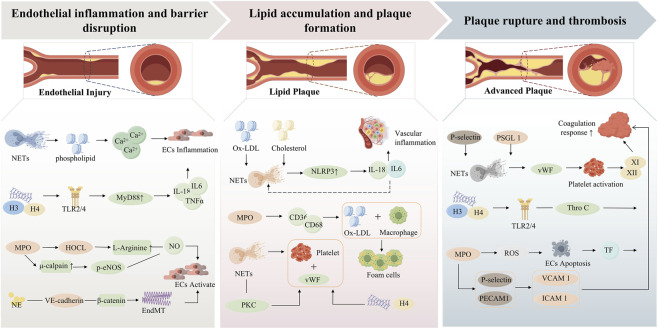
Schematic illustration of the mechanisms by which NETs promote atherosclerosis progression.

The pathological progression of atherosclerosis is generally divided into three stages: fatty streak formation, plaque progression, and thrombotic complications. Recent evidence indicates that NETs are involved not only in autoimmune diseases and infections but also in the onset and progression of atherosclerosis. The proposed mechanisms are summarized in Figure 1.

The vascular endothelium is composed of endothelial cells (ECs), which are essential for maintaining an anti-adhesive, profibrinolytic, and anti-inflammatory state (Jebari-Benslaiman et al., 2022; Landmesser et al., 2004). Endothelial dysfunction is an early event in atherosclerosis, and accumulating evidence indicates that NETs contribute to this process (Warnatsch et al., 2015). Histones released from NETs can bind to endothelial phospholipids, increase membrane permeability, promote Ca^2+^ influx, and induce cell injury or death (Morales-Primo et al., 2022). In addition, histones H3 and H4 downregulate junction-related proteins, including vascular endothelial cadherin (VE-cadherin), p120-catenin, and zonula occludens-1 (ZO-1), through Toll-like receptor 4 (TLR4)-dependent signaling. This disruption destabilizes endothelial junctions and increases vascular permeability. Notably, pharmacological inhibition can partially attenuate histone-induced hyperpermeability (Ramasubramanian et al., 2022). MPO is another important mediator of NET-induced endothelial injury. Hypochlorous acid (HOCl), generated by MPO, reduces nitric oxide (NO) bioavailability by modifying L-arginine-related pathways (Zhang et al., 2001). MPO also enhances leukocyte-endothelial adhesion through μ-calpain activation, promotes endothelial nitric oxide synthase (eNOS) dysregulation, and further decreases NO levels, thereby aggravating endothelial dysfunction (Etwebi et al., 2018).

Lipid deposition within the arterial wall is driven largely by oxLDL. Oxidized low-density lipoprotein promotes lipoprotein aggregation, endothelial injury, leukocyte recruitment, foam cell formation, and vascular inflammation (Malekmohammad et al., 2021; Li et al., 2021). Moreover, increased oxLDL uptake together with impaired cholesterol efflux may synergistically enhance NET-mediated activation of the NLR family pyrin domain containing 3 (NLRP3) inflammasome, thereby promoting the release of the proinflammatory cytokines interleukin (IL)-1β and IL-18 (Sheedy et al., 2013). IL-18 can further stimulate NET formation, thus creating a positive feedback loop (Kahlenberg et al., 2013). MPO associated with NETs also promotes ROS production and facilitates LDL oxidation, which further enhances foam cell formation (Tangeten et al., 2022). In parallel, MPO can increase the expression of scavenger receptors such as CD36 and CD68 on macrophages, thereby promoting oxLDL uptake and accelerating plaque growth (Abdo et al., 2017; Quinn et al., 2011). In addition, NETs may promote platelet adhesion and microthrombus formation through interactions with von Willebrand factor (vWF).

Plaque rupture, erosion, and thrombosis are major complications of advanced atherosclerosis and are key triggers of acute coronary syndromes. In this context, platelets function not only as mediators of hemostasis but also as active participants in vascular inflammation (Martinez Bravo et al., 2024). Activated platelets interact with neutrophils through P-selectin binding to P-selectin glycoprotein ligand-1 (PSGL-1), thereby promoting NET formation. In addition, platelet-derived vWF cooperates with NETs to enhance platelet adhesion, aggregation, fibrin formation, and immunothrombosis (Carestia et al., 2016; Fuchs et al., 2010). Histones within NETs can also promote platelet activation and thrombin generation through TLR2 and TLR4 signaling, while impairing anticoagulant activity by inhibiting thrombomodulin-dependent protein C activation (Ammollo et al., 2011). Furthermore, NET-associated cathepsin G (CG) and NE enhance coagulation by modulating platelet surface molecules and degrading endogenous anticoagulants. At the same time, the DNA scaffold of NETs provides a structural framework for platelet and erythrocyte binding, which supports thrombus growth (Cerletti et al., 1995). NETs may also amplify thrombin generation through activation of platelets and coagulation factors XI and XII. In parallel, they activate endothelial cells and promote the release of proinflammatory mediators such as IL-8 and IL-1β, thereby further accelerating thrombus formation (Gupta et al., 2010).

## 5 Potential of plant-derived metabolites to modulate NETs for atherosclerosis treatment

Before discussing specific compounds, two principles should be clarified. First, statistically significant changes in NET-related biomarkers under experimental conditions do not necessarily indicate genuine clinical benefit in human atherosclerosis (Alp et al., 2025)**.** Second, for polyphenols or other metabolites with strong redox activity, an apparent “anti-NET” effect observed in assays based on fluorescence, ROS, or extracellular DNA requires further validation using orthogonal methods. This step is necessary to exclude assay interference and non-specific broad-spectrum effects (Bisson et al., 2016)**.** Detailed information on the potential of plant-derived metabolites to modulate NETs for atherosclerosis treatment is provided in Table 1.

**TABLE 1:** Plant-derived metabolites targeting NETs for the treatment of atherosclerosis.

Category	References	Plant-derived metabolites	Molecular formula	In vivo/In vitro/Clinical trial	Model	Dose and duration	Control group	Positive control drug	Outcomes
Alkaloids	Li et al. (2024)	Colchicine	C_22_H_25_NO_6_	*In vivo*	Male C57BL/6 mice + LAD ligation	0.1 mg/kg, intraperitoneal injection, 1 week	Sham group; AMI model group	Cl-amidine 10 mg/kg/d	NETs↓, CitH3↓, Ly6G↓, ROS↓, gp91phox (NOX2)↓, Ca2+ influx↓, p-CaMKII↓, TNF-α↓, IL-6↓, IL-1β↓, caspase-1 p20↓, IL-1β p17↓, NLRP3↓, α-SMA↓, TUNEL↓, macrophage infiltration↓, survival rate↑, cardiac function↑, myocardial fibrosis↓
				*In vitro*	Bone marrow neutrophils from male C57BL/6 mice + PMA	5 nM, PMA stimulation for 3.5 h after pretreatment	Unstimulated control; PMA-stimulated group	Cl-amidine	NETs↓, CitH3↓, ROS↓, Ca2+ influx↓, p-CaMKII↓
	Vaidya et al. (2021)	Colchicine	C_22_H_25_NO_6_	Clinical trial	Patients with acute coronary syndrome undergoing PCI	1.5 mg, orally, administered before PCI	Stable angina control; untreated ACS control	None	NETs↓, NE↓, MPO↓, chromatin swelling↓, disruption of α-tubulin microtubule cytoskeletal rearrangement↓, MTOC↓
				*In vitro*	Human neutrophils + PMA/LPS/IL-8/L. amazonensis	25 nmol/L, pretreated for 1.5 h	Unstimulated control; stimulated control	None	NETs↓, NE↓, MPO↓, chromatin swelling↓, disruption of α-tubulin microtubule cytoskeletal rearrangement↓, MTOC↓
Terpenoids	Yang et al. (2025)	Ginsenoside Rb1	C_54_H_92_O_23_	*In vivo*	HFD-fed Apoe−/− mice	15, 60 mg/kg, intraperitoneal injection, 8 weeks	Normal diet group; HFD model group	None	m6A methylation↑, FTO↓, ICAM1↓, VCAM1↓, SELE↓, SELP↓, neutrophil adhesion↓, THP-1 adhesion↓, TEER↑, Evans blue permeability↓, endothelial barrier function↑, plaque area↓, lipid deposition↓
				*In vitro*	HAECs exposed to NETs	Rb1 100 μM, pretreated for 30 min; NETs 1 μg/mL for 12–24 h	Unstimulated control; NET-stimulated group	None	m6A methylation↑, FTO↓, ICAM1↓, VCAM1↓, SELE↓, SELP↓, neutrophil adhesion↓, THP-1 adhesion↓, TEER↑, Evans blue permeability↓, endothelial barrier function↑
	Song et al. (2023)	Celastrol	C_29_H_38_O_4_	*In vitro*	Peripheral blood neutrophils from healthy donors + PMA	0.3125, 0.625, 1.25 μM, pretreated for 30 min followed by PMA stimulation for 3 h	Unstimulated control; PMA-stimulated group	None	NETs↓, Sytox Green fluorescence↓, ROS↓, no significant change in cytotoxicity
Flavonoids	Liu et al. (2024)	Quercetin	C_15_H_10_O_7_	*In vitro*	Primary rat neutrophils + LPC	25 μmol/L for 3 h	Unstimulated control; LPC-stimulated group	None	NETs↓, H3Cit↓, MPO-DNA↓, cf-DNA↓, P2X7R↓, p-P38MAPK↓, NOX2↓
	Goshovska et al. (2025)	Quercetin	C_15_H_1_0O_7_	*In vivo*	Wistar rat LAD ischemia/reperfusion model	50 mg/kg, tail vein injection, administered 10 min before reperfusion	Sham group; MI/R model group	None	NETs↓, myocardial injury↓, infarct size↓, left ventricular systolic function↑, hemodynamic improvement↑
				*In vitro*	Rat neutrophils + PMA	Quercetin dose not clearly specified in the article; PMA 20 nM stimulation for 3 h	Unstimulated control; PMA-stimulated group	None	NETs↓
	Ohinata et al. (2024)	Catechin	C_15_H_14_O_6_	*In vitro*	dHL-60 neutrophil-like cells + PMA/POVPC	Compound dose not uniformly reported in the article; pretreated for 30 min followed by stimulation for 2 h	Unstimulated control; PMA/POVPC-stimulated group	None	NETs↓, extracellular DNA release↓, PMA-induced NETosis↓, POVPC-induced NETosis↓
	Juanlu et al. (2026)	Hesperidin/Baicalin	C_28_H_34_O_15_/C_21_H_18_O_11_	*In vitro*	dHL-60 NETosis screening model + PMA	10 nM each, pretreated for 1 h followed by PMA 200 nM stimulation for 4 h	DMSO control; PMA-stimulated group	None	NETosis↓, extracellular DNA release↓, TREM1↓, S100A8↓, S100A9↓, CCL7↓, PADI4↓, thrombin activity↓
	Cai et al. (2025)	Baicalin	C_21_H_18_O_11_	*In vivo*	STZ-induced hyperglycemic mice	240, 1,200 mg/kg, gavage, 2 weeks	Normal group; STZ model group	DNase I 2.5, 10 mg/kg	NETs↓, H3Cit↓, intestinal epithelial injury↓, loss of tight junction proteins↓, cell viability↑, intestinal barrier function↑
				*In vitro*	Mouse neutrophils + high glucose/LPS; IEC-6 stimulated with NETs	25, 50, 100 μM for 2.5 h; NET stimulation of IEC-6 for 12 h	Low-glucose control; high glucose/LPS-stimulated group; NET-stimulated IEC-6 model group	DNase I	NETs↓, H3Cit↓, IEC-6 viability↑, loss of tight junction proteins↓, barrier injury↓
	Tang et al. (2025)	Formononetin	C_16_H_12_O_4_	*In vivo*	SD rat MI/R model	10, 20, 40 mg/kg, gavage, 1 week; final dose administered 60 min before modeling	Sham group; MI/R model group	Diltiazem 16 mg/kg	Infarct size↓, LVEF↑, LVFS↑, platelet aggregation↓, platelet adhesion↓, granule release↓, platelet–leukocyte aggregation↓, microthrombosis↓, NETs↓, CD36↓, p-ERK5↓
				*In vitro*	Coculture of normal rat platelets and sorted neutrophils	40 μM, coculture for 1 h	Unactivated control; Trap-6-activated group	SSO 200 μM	Platelet activation↓, platelet–neutrophil interaction↓, NETs↓, CD36↓, p-ERK5↓
Phenolics	Ohinata et al. (2024)	Resveratrol	C_14_H_12_O_3_	*In vitro*	dHL-60 neutrophil-like cells + PMA/POVPC	Compound dose not uniformly reported in the article; pretreated for 30 min followed by stimulation for 2 h	Unstimulated control; PMA/POVPC-stimulated group	None	NETs↓, extracellular DNA release↓, PMA-induced NETosis↓, POVPC-induced NETosis↓
	de Mattos et al. (2024)	Resveratrol	C_14_H_12_O_3_	*In vitro*	Human neutrophils + PMA/LPS/IL-8/Leishmania	25, 50, 100 μM, pretreated for 30 min followed by stimulation for 1–4 h	Unstimulated control; PMA/LPS/IL-8/Leishmania-stimulated group	None	NET-DNA release↓, H2O2↓, MPO activity↓, NE nuclear translocation↓
	Zhou et al. (2024)	Sodium ferulate	C_10_H_9_NaO_4_	*In vivo*	MPO-AAV model in male Rag2−/− mice	100 mg/kg, intraperitoneal injection, 10 days	Normal group; MPO-AAV model group	None	NETs↓, CitH3↓, MPO↓, CD62p↓, PAC-1↓, β-TG↓, sTM↓, iNOS↓, TNF-α↓, vWF↓, ANCA↓, MTT activity↑, DNA release↓
				*In vitro*	EPCs derived from patients with AAV/model stimulation	100, 200, 400 ng/mL for 24 h	Normal stimulation control; model stimulation group	None	DNA release↓, EPC viability↑, β-TG↓, iNOS↓, endothelial injury↓
	Ma et al. (2025)	Paeonol	C_9_H_10_O_3_	*In vivo*	Male ApoE−/− mice + HFD	200, 400 mg/kg, gavage, 4 weeks	Normal diet group; HFD model group	Simvastatin 5 mg/kg	NETs↓, NE↓, MPO↓, CitH3↓, caspase-1↓, foam cell inflammation↓, NLRP3↓, TNF-α↓, IL-1β↓, IL-18↓, atherosclerotic plaque burden↓, plaque area↓
				*In vitro*	dHL-60-derived neutrophils; coculture of NETs and RAW264.7 foam cells	15–240 μM for 4 h	Unstimulated control; PMA-stimulated group; foam cell model group	None	NETs↓, NE↓, CitH3↓, NLRP3↓, caspase-1↓, TNF-α↓, IL-1β↓, IL-18↓, foam cell inflammation↓

### 5.1 Alkaloids

Colchicine is a widely used anti-inflammatory alkaloid (Wołowiec et al., 2025) that is mainly derived from *Colchicum autumnale* L [Colchicaceae] and related species (Deftereos et al., 2022). Recent large-scale meta-analyses have shown that colchicine has preventive and therapeutic value in myocardial infarction, ischemic stroke, and coronary artery disease (Samuel et al., 2025; Martí-Carvajal et al., 2025; Laudani et al., 2026). In a C57BL/6 mouse model of acute myocardial infarction (AMI), Li and colleagues (Li et al., 2024) showed that colchicine reduced myocardial Ly6G^+^ neutrophil infiltration and the abundance of citrullinated histone H3 (CitH3)^+^ NETs. These findings suggest that colchicine suppresses NET formation and attenuates inflammation. Mechanistically, this effect may involve inhibition of NOX2 upregulation, reduced ROS production, suppression of CaMKII phosphorylation, and decreased Ca^2+^ influx. Together, these changes may limit ROS/Ca^2+^-dependent chromatin decondensation and NET release.

Similar translationally relevant observations have been reported in humans. Vaidya and colleagues (Vaidya et al., 2021) reported that, in patients undergoing percutaneous coronary intervention (PCI), oral colchicine reduced the local coronary sinus release of NETs and NE. Their *ex vivo* experiments showed that colchicine did not significantly affect PMA-induced ROS production, but it reduced chromatin swelling and restored the structural organization of α-tubulin and the microtubule-organizing center (MTOC). These findings suggest that cytoskeletal stabilization limits nuclear expansion and membrane rupture. However, this study had a modest sample size and focused on short-term biomarker release rather than plaque biology or hard cardiovascular outcomes. Therefore, NET suppression should not be interpreted as definitive evidence of clinical efficacy.

### 5.2 Terpenoids

Ginsenoside Rb1 is a monomeric triterpenoid saponin isolated from *Panax ginseng* C.A.Mey [Araliaceae]. It exhibits a broad range of biological activities, with well-documented anti-inflammatory effects (Zhuang et al., 2025). Yang (Yang et al., 2025) used a high-fat diet (HFD)-fed Apoe^−/−^ mouse model and NET-exposed human aortic endothelial cells (HAECs). Their study showed that Rb1 attenuated endothelial activation, reduced monocyte adhesion, and decreased plaque burden. Mechanistically, the authors proposed that Rb1 suppressed aberrant activation of fat mass and obesity-associated protein (FTO), preserved the N6-methyladenosine (m6A) status and stability of TNIP1 mRNA, and thereby limited nuclear factor kappa B (NF-κB) activation and the expression of adhesion molecules, including ICAM-1 and VCAM-1. These findings are relevant because the *in vivo* model reflects key features of atherosclerosis. However, several limitations should be noted. The cellular experiments primarily assessed endothelial responses to exogenously prepared NETs rather than direct inhibition of NET formation. The *in vitro* concentration (100 μM) is relatively high and may limit pharmacological relevance. In addition, the study did not include human NET-related biomarkers or clinical outcome data. Therefore, although the HAEC findings provide mechanistic insight, they do not establish clinical efficacy in humans.

Celastrol, another bioactive terpenoid derived from *Tripterygium wilfordii* Hook. f. [Celastraceae] (Li et al., 2022). It also shows inhibitory effects on NET-related readouts. Song et al. (Song et al., 2023) conducted *in vitro* experiments and showed that celastrol did not cause significant cytotoxicity in human peripheral blood neutrophils at concentrations of 0.3125–1.25 μM. They also found that celastrol reduced PMA-induced NET release and intracellular ROS production. These findings are consistent with inhibition of ROS-dependent NET formation. Therefore, its inhibitory effects may not be specific to NET formation. In addition, the study mainly relied on PMA stimulation and fluorescence-based NET assays. It did not include *in vivo* validation in atherosclerosis models or orthogonal counterscreens to exclude nonspecific interference. Therefore, the current evidence should be regarded as mechanistic and does not establish clinical efficacy in humans.

### 5.3 Flavonoids

Quercetin is a common dietary flavonoid that is widely found in onions, apples, grapes, and other plant-derived foods. It exhibits antioxidant, anti-inflammatory, and antithrombotic activities (Ozorowski et al., 2025). In atherosclerosis, lysophosphatidylcholine (LPC), an important component of oxLDL, can induce NETs formation. This process promotes the release of cell-free DNA (cfDNA), MPO-DNA complexes, and citrullinated histone H3 (H3Cit), thereby amplifying local inflammation and contributing to plaque instability. Liu et al. (Liu et al., 2024) established an LPC-induced NET model in primary neutrophils and showed that quercetin downregulated P2X7R and inhibited the p38 MAPK/NOX2 pathway. As a result, levels of H3Cit, MPO, cfDNA, and MPO-DNA were significantly reduced. Moreover, BzATP or a p38 agonist partially reversed the inhibitory effect of quercetin, suggesting that quercetin suppresses NET formation, at least in part, through the P2X7R/p38 MAPK/NOX2 axis. In another study, water-soluble quercetin was administered intravenously in a rat model of myocardial infarction (Goshovska et al., 2025). This treatment reduced peripheral NET formation and plasma free DNA levels, while improving cardiac function. These findings are biologically interesting, but they require caution. Quercetin is a redox-active flavonoid with well-recognized potential for assay interference (Bisson et al., 2016; Baell, 2016). In addition, the *in vitro* work relied heavily on extracellular DNA- and reactive oxygen species-linked readouts, and the myocardial ischemia-reperfusion model is not equivalent to chronic atherosclerosis. Moreover, the direct anti-NET mechanism remains incompletely distinguished from broader antioxidant or cytoprotective effects, and the human exposure profile is dominated by extensive metabolism rather than sustained free aglycone exposure (Burak et al., 2017; Zhu et al., 2024). Accordingly, these *in vitro* and animal findings are hypothesis-generating and do not demonstrate efficacy in humans.

Catechin is naturally present in various foods and beverages, including tea, berries, grapes, and wine. It has antioxidant properties and can scavenge reactive oxygen species, reduce free radical generation, and limit lipid peroxidation (Farhan, 2022; Musial et al., 2020). Ohinata et al. (Ohinata et al., 2024) used differentiated HL-60 (dHL-60) neutrophil-like cells and found that catechin pretreatment inhibited PMA-induced DNA extrusion and MPO release, including under co-stimulation with the oxidized phospholipid 1-palmitoyl-2-(5-oxovaleroyl)-sn-glycero-3-phosphocholine (POVPC). Structure–activity analysis suggested that planarized catechin analogs exerted stronger inhibitory effects.

Using gene set enrichment analysis and a PMA-induced dHL-60 cell model, Juanlu et al. (Juanlu et al., 2026) found that hesperidin and baicalin significantly reduced the proportion of morphologically NETosis-positive cells and extracellular DNA exposure. These compounds also downregulated NET-related genes, including TREM1, S100A8, S100A9, and CCL7. In addition, both compounds reduced thrombin activity in human plasma exposed to NET-containing culture medium, suggesting that they may not only inhibit NET formation but also attenuate NET-associated procoagulant activity. Cai et al. (Cai et al., 2025) further reported that baicalin reduced H3Cit levels and NET formation in high-glucose/LPS-stimulated mouse neutrophils. The authors also found that baicalin partially alleviated intestinal epithelial barrier injury in STZ-induced hyperglycemic mice. These studies extended the discussion from screening observations to disease-associated inflammation. However, several important limitations remained. The hesperidin/baicalin study relied on dHL-60 screening conditions and did not include primary human neutrophils or *in vivo* validation in atherosclerosis models. In contrast, the baicalin study used a diabetic intestinal barrier model that was only indirectly related to plaque biology and employed very high oral doses. Therefore, although both studies reported statistically significant changes in NET-related readouts, these findings did not establish clinically relevant benefit in human atherosclerosis. Likewise, the *in vitro* observations did not demonstrate efficacy in humans.

Formononetin, an isoflavone, has also attracted attention for its potential role in NET-associated thromboinflammation (Machado Dutra et al., 2021). Tang et al. (Tang et al., 2025) reported in a rat myocardial ischemia-reperfusion model that formononetin reduced infarct size, improved cardiac function, inhibited platelet activation and platelet–leukocyte aggregation, and decreased myocardial NET deposition. In a platelet-neutrophil coculture system, formononetin also suppressed NET formation induced by activated platelets and was associated with reduced CD36 expression and ERK5 phosphorylation. These findings suggest that formononetin may modulate platelet-driven secondary NET formation in an acute ischemic setting. However, several limitations should be noted. The evidence was derived from myocardial ischemia-reperfusion and coculture models rather than from models of chronic plaque progression. In addition, the *in vitro* concentration was relatively high, and the comparator drug was not NET-specific. Therefore, these preclinical data do not establish efficacy in humans or direct anti-atherosclerotic activity.

### 5.4 Other polyphenols

Resveratrol is a representative non-flavonoid polyphenol of the stilbene class. It is widely present in plants such as grapes and *Reynoutria japonica Houtt.* [syn: *Polygonum cuspidatum Siebold* & Zucc. Polygonaceae] and has antioxidant, anti-inflammatory, and cardioprotective activities (Brown et al., 2024). Ohinata et al. (Ohinata et al., 2024) established a NET model using differentiated HL-60 (dHL-60) cells and found that resveratrol pretreatment dose-dependently inhibited PMA-induced DNA extrusion. Under co-stimulation with the oxidized phospholipid POVPC and PMA, resveratrol still significantly reduced NET formation and MPO release. These findings suggest that resveratrol not only suppresses NET formation induced by classical stimuli, but also counteracts the enhancement of NET release driven by oxidized lipids. Another study (de Mattos et al., 2024) reported that resveratrol inhibited NET release induced by PMA, LPS, IL-8, and Leishmania. This effect was accompanied by lower H2O2 levels, reduced MPO activity, and inhibition of NE nuclear translocation. These results suggest that resveratrol may act across multiple stimuli. Nevertheless, several limitations should be considered. Resveratrol is a classic redox-active polyphenol with substantial potential for assay interference. In addition, the dHL-60 study used a surrogate cell system, and the primary neutrophil study employed concentrations of 25–100 μM. These concentrations are difficult to reconcile with the low free systemic exposure observed after oral administration in humans (Mohammadipoor et al., 2022; Szymkowiak et al., 2025). Therefore, the *in vitro* findings do not establish efficacy in humans and should not be overinterpreted as evidence of specific NET targeting.

Sodium ferulate is a phenolic compound widely distributed in plants. It is particularly abundant in the cell walls of fruits, vegetables, and grains, as well as in the seeds, leaves, and roots of cereals (Purushothaman and Rizwanullah, 2024). Sodium ferulate has antioxidant, antibacterial, and anti-inflammatory properties (Liu et al., 2025). Because vascular inflammation is a central process in atherosclerosis (Libby and Soehnlein, 2025), agents that suppress inflammatory amplification may have therapeutic potential. Zhou et al. (Zhou et al., 2024) reported, based on *in vitro* and *in vivo* experiments, that sodium ferulate inhibited NET-associated vascular inflammation, downregulated platelet activation markers such as CD62P and CD63, reduced circulating CitH3 and MPO levels, and preserved endothelial barrier integrity. However, several limitations should be noted. The *in vivo* model was myeloperoxidase-ANCA-associated vasculitis in Rag2^−/−^ mice rather than atherosclerosis. In addition, the cellular experiments used EPC-related injury models from patients with vasculitis rather than plaque-relevant vascular tissues. Therefore, these data support possible anti-inflammatory activity in NET-rich vascular injury, but they do not demonstrate efficacy in human atherosclerosis. Likewise, the *in vitro* findings should not be interpreted as evidence of clinical benefit.

Paeonol is a major bioactive component of the root bark of *Paeonia × suffruticosa* Andrews [Paeoniaceae] and exhibits anti-inflammatory, antitumor, and antifibrotic activities (Shu et al., 2026). Ma et al. (Ma et al., 2025) reported that, in HFD-fed ApoE−/− mice, paeonol reduced NET formation, attenuated CitH3/NLRP3/caspase-1 signaling in foam cells, and decreased aortic plaque area. This disease model gives the study greater relevance to atherosclerosis than many other reports in this field. However, several limitations should be considered. The mechanistic experiments relied on dHL-60-derived neutrophils and RAW264.7 foam cells. In addition, the *in vivo* doses were high, and the study did not demonstrate direct target engagement within the NET pathway. Therefore, the cell-based findings do not establish efficacy in humans, and the overall evidence remains preclinical.

## 6 Specificity concerns in studies of plant-derived NET modulators

Although some plant-derived metabolites reduce NET-related assay readouts in experimental systems, these findings do not necessarily indicate selective NET inhibition or therapeutic potential in humans. A major pharmacological concern is assay interference. Many plant-derived metabolites, especially flavonoids and other polyphenolic compounds, contain redox-active or highly conjugated structural units. These structures can generate false-positive signals through redox cycling, covalent reactions after quinone formation, colloidal aggregation, metal ion chelation, fluorescence emission or quenching, light scattering, membrane disruption, or nonspecific protein binding (Tan et al., 2024). This issue is particularly important in NET research because common endpoints, such as Sytox/PicoGreen fluorescence, extracellular DNA release, MPO activity, ROS production, H3Cit immunostaining, and loss of endothelial barrier function after exposure to NET-rich preparations, may be affected by general antioxidant effects, dye interference, or changes in cell viability rather than by direct modulation of the NET program itself. Therefore, the NET-inhibitory activity of plant-derived metabolites should be evaluated using pharmacological criteria that are more rigorous than mere statistical significance (Magalhães et al., 2021; Bolz et al., 2021).

In the relevant literature, the distinction between statistical significance and clinical relevance is especially important. Under strictly controlled *in vitro* conditions, a compound may cause a nominally significant reduction in cfDNA, MPO-DNA, H3Cit, or ROS levels. However, this finding does not mean that the compound can prevent plaque progression, plaque rupture, myocardial infarction, or stroke in humans (Alp et al., 2025). Although *in vitro* studies can clarify mechanisms under controlled conditions, they do not account for human pharmacokinetics, metabolism, protein binding, drug exposure at plaque sites, comorbidities, or safety risks. This limitation is particularly evident when aglycone polyphenols are tested at concentrations in the tens of micromoles. Such concentrations may not reflect actual exposure levels in human circulation and do not account for the predominance of conjugated metabolites after oral administration (Burak et al., 2017; Szymkowiak et al., 2025; Zhu et al., 2024). Therefore, claims about treatment outcomes should be based on the overall strength of the evidence rather than on p-values alone.

As shown in Table 2, the translational credibility of the included studies is heterogeneous. Among the metabolites discussed in this mini-review, colchicine is supported by comparatively stronger evidence. In addition to its effects on chromatin swelling and cytoskeletal organization *in vitro*, colchicine has shown NET-related activity in both animal studies of myocardial infarction and clinical studies in patients undergoing PCI (Vaidya et al., 2021; Li et al., 2024). Therefore, colchicine currently represents one of the most translationally relevant examples of NET modulation. In contrast, plant-derived metabolites such as quercetin, resveratrol, catechin, and hesperidin require more cautious interpretation. Many of these flavonoids and polyphenolic metabolites have marked redox activity and broad protein-binding capacity. As a result, they may alter NET-related readouts without directly inhibiting NETosis (Baell, 2016). In addition, most available studies rely on PMA- or LPC-induced systems, differentiated HL-60 surrogate cells, or assays based on fluorescence signals and extracellular DNA release. Each of these approaches has inherent limitations. Therefore, a reduction in experimental readouts may reflect decreased oxidative stress or interference with the detection system rather than specific suppression of NET formation. Differentiated HL-60 cells can be useful for preliminary or high-throughput screening, but their NET-related responses are generally weaker and less stable than those of primary neutrophils (Malavez-Cajigas et al., 2024). Accordingly, results obtained from dHL-60 models should be regarded as supportive screening data rather than definitive evidence of specific anti-NET activity. A similar caution applies to celastrol. Although celastrol reduces ROS levels and NET-related release at low micromolar concentrations, it is a highly reactive triterpenoid metabolite with broad stress-response activity. At present, it is more appropriate to describe celastrol as a modulator of NET-related phenotypes than as a confirmed specific NET inhibitor. Nevertheless, limited specificity *in vitro* does not mean that these metabolites lack all human evidence. Quercetin has shown effects on blood pressure and selected metabolic markers in several randomized controlled trials and meta-analyses (Popiolek-Kalisz and Fornal, 2022; Serban et al., 2016). These findings support its human bioavailability and biological activity, but they do not demonstrate direct NET-targeting specificity. Resveratrol has also been evaluated in multiple randomized controlled trials in populations with metabolic disorders or elevated cardiovascular risk (Mohammadipoor et al., 2022). These studies suggest potential benefits for endothelial function and inflammatory markers, but they mainly support general vasoprotective or metabolic effects rather than confirmed anti-NET activity in humans. Hesperidin and baicalin also have some clinical or translational support, but the primary endpoints in relevant studies have mostly focused on inflammation, metabolism, or vascular function rather than NET biomarkers (Lorzadeh et al., 2019; Khorasanian et al., 2023). Human evidence for ginsenoside Rb1 currently derives mainly from tolerability and pharmacokinetic studies of red ginseng-containing formulations rather than from intervention trials assessing disease-specific outcomes or NET-related endpoints. Therefore, Rb1 is better regarded at present as a candidate metabolite supported by human exposure data rather than by direct efficacy evidence (Choi et al., 2020). Similar limitations apply to paeonol, formononetin, and sodium ferulate. Although animal studies and pathology-related models provide preliminary support, randomized controlled trials in humans that address NET-related outcomes remain lacking (Shen et al., 2023; Zhang et al., 2019; Sharma and Kabra, 2025). Taken together, these plant-derived metabolites should currently be viewed as candidates with translational potential rather than as fully validated and specific NET inhibitors.

**TABLE 2:** Critical appraisal of the 14 included studies summarized in this mini-review.

References	Plant-derived metabolites	Experimental context	Major limitations affecting specificity/translation	Bottom-line interpretation
Li et al. (2024)	Colchicine	Male C57BL/6 AMI model; PMA-stimulated marrow neutrophils	Acute infarction/remodeling rather than chronic plaque biology; male-only design; NET reduction may reflect broad anti-inflammatory action	Supports concept of NET modulation, not specific anti-atherosclerotic efficacy
Vaidya et al. (2021)	Colchicine	ACS patients undergoing PCI; *ex vivo* neutrophils	Human mechanistic study, but modest size, acute biomarker endpoints, and no proof that NET lowering mediated long-term clinical benefit	Most translationally relevant entry, but still mechanistic rather than definitive efficacy evidence
Yang et al. (2025)	Ginsenoside Rb1	HFD-fed Apoe−/− mice; HAECs exposed to NETs	Atherosclerosis-relevant mouse model, but endothelial-cell arm used 100 μM and assessed NET-exposed ECs rather than direct NET inhibition; no human NET endpoints	Promising preclinical candidate, but human efficacy is unproven
Song et al. (2023)	Celastrol	Primary human neutrophils + PMA	Single *in vitro* model; ROS/Sytox-dominant readouts; chemically reactive scaffold; no orthogonal counterscreens or *in vivo* atherosclerosis validation	Mechanistic signal only; specificity remains uncertain
Liu et al. (2024)	Quercetin	Primary rat neutrophils + LPC	Redox-active flavonoid; reliance on cfDNA/MPO-DNA/H3Cit/ROS-linked readouts; no orthogonal interference controls; rat cells only	Hypothesis-generating, with substantial PAINS-like uncertainty
Goshovska et al. (2025)	Quercetin	Rat ischemia/reperfusion model; PMA *in vitro*	Not a chronic atherosclerosis model; IV pretreatment limits relevance; *in vitro* dose was not clearly reported; benefit may be secondary to smaller infarction	Does not establish direct anti-NET or anti-atherosclerotic efficacy
Ohinata et al. (2024)	Catechin	dHL-60 cells + PMA/POVPC	Surrogate cell line; dose reporting incomplete; fluorescence/DNA extrusion endpoints are vulnerable to polyphenol-related interference	Screening-level evidence only
Juanlu et al. (2026)	Hesperidin/Baicalin	dHL-60 PM A screening model	Single screening context without primary human neutrophils, animal atherosclerosis validation, or pharmacokinetic grounding	Best interpreted as early screening data
Cai et al. (2025)	Baicalin	STZ mice; mouse neutrophils + high glucose/LPS	Disease context is intestinal barrier injury rather than plaque biology; very high oral doses; *in vitro* concentrations pharmacologically demanding	Indirect relevance to atherosclerosis; not human efficacy evidence
Tang et al. (2025)	Formononetin	Rat MI/R model; platelet-neutrophil coculture	Acute thromboinflammation model, not chronic plaque evolution; 40 μM *in vitro*; comparator not NET-specific	Mechanistically interesting but translationally indirect for atherosclerosis
Ohinata et al. (2024)	Resveratrol	dHL-60 cells + PMA/POVPC	Same surrogate-cell and fluorescence-readout limitations as catechin, with dose reporting not fully standardized	Screening-level evidence only
de Mattos et al. (2024)	Resveratrol	Primary human neutrophils + PMA/LPS/IL-8/Leishmania	Broad antioxidant polyphenol tested at 25–100 μM; atherosclerosis specificity and orthogonal interference controls are lacking	Primary-cell data are useful, but human efficacy is not shown
Zhou et al. (2024)	Sodium ferulate	MPO-AAV vasculitis model in Rag2−/− mice; EPC injury assays	Disease mismatch with atherosclerosis; immunodeficient host; human cellular material derived from vasculitis rather than plaque biology	Supports vascular anti-inflammatory activity, not direct anti-atherosclerotic efficacy
Ma et al. (2025)	Paeonol	HFD-fed ApoE−/− mice; dHL-60/RAW264.7 mechanistic assays	Relevant mouse model, but high doses, surrogate cell systems, and no direct target-engagement or human data	Preclinical support is stronger than for many polyphenols, yet still insufficient for human efficacy claims

## 7 Conclusion and prospects

In summary, NETs contribute to multiple stages of atherosclerosis. Key NET-associated effectors, including histones and MPO, promote endothelial activation and upregulate adhesion molecules. These changes amplify local inflammation and accelerate plaque progression. In advanced lesions, NETs also provide a structural scaffold for thrombus formation and disrupt coagulation homeostasis. As a result, NETs may increase the risk of plaque erosion, plaque rupture, and thrombotic events. Current studies suggest that some plant-derived metabolites can modulate NET formation, persistence, or clearance through multiple pathways and targets. Through these effects, these metabolites may influence vascular inflammation, plaque development, and thrombosis. However, the available evidence remains preliminary. Therefore, targeting NETs with plant-derived metabolites should be regarded as a promising but not yet clinically validated strategy for atherosclerosis.

The major limitations are also clear. First, NET biology is heterogeneous. Different stimuli, tissue compartments, and disease stages likely generate functionally distinct NET programs. Second, many studies rely on acute injury models or non-atherosclerotic models, surrogate cell systems such as dHL-60 cells, or stimulation protocols dominated by PMA, LPC, LPS, or high glucose. Third, for polyphenols and flavonoids, PAINS-like interference and other nonspecific effects can confound NET readouts, especially when studies rely on a single fluorescence-based assay or extracellular DNA-based assay. Fourth, many studies do not adequately address pharmacokinetic realism. Concentrations that appear effective *in vitro* may not be achievable clinically at the lesion site. Even when such exposure is achievable, the active circulating species in humans may differ from the tested aglycone. Finally, NET suppression is not inherently benign because indiscriminate inhibition may compromise antimicrobial defense.

Accordingly, future research should emphasize rigor over enthusiasm.Candidate compounds should be prioritized only after confirmation by orthogonal NET assays, demonstration of pharmacokinetically plausible exposure, use of primary human neutrophils and atherosclerosis-relevant *in vivo* models, and clear separation of direct NET inhibition from secondary antioxidant or anti-inflammatory effects. Early translational studies should incorporate circulating NET biomarkers, imaging endpoints or plaque-relevant endpoints, and careful safety monitoring. These measures are necessary to distinguish statistically significant biomarker changes from clinically meaningful benefit. Based on the current evidence, plant-derived metabolites should be regarded as promising leads for further investigation rather than as clinically validated NET-targeted therapies for atherosclerosis.
